# Rethinking Breast Self-Examinations: Are We Asking the Right Questions?

**DOI:** 10.4137/bcbcr.s786

**Published:** 2008-06-17

**Authors:** Steven J. Danish, Suzzette M. Chopin, Kathryn Conley

**Affiliations:** Life Skills Center, Department of Psychology, Virginia Commonwealth University

The debate over the efficacy of breast self-exams (BSE) tends to be couched in economic terms based on data aggregation. There are conflicting reports of its effectiveness as well as its overall costs to the health care system. Researchers and most funding agencies suggest it is not a useful tool in cancer prevention and often drives up health care costs because of the number of false positives. On the other hand, many physicians still recommend it because their patients have detected lumps through self-exams.

A case–control study within the Canadian National Breast Screening suggested that the use of certain BSE techniques may lead to fewer breast cancer deaths (Harvey, Miller, Baines, and Corey, 1997). A later meta-analysis by Canadian researchers found no benefit for BSEs in any age group and resulted in the Canadian Task Force on Preventive Health Care recommending against teaching BSE. (Baxter et al). The authors did recommend further research be conducted on BSEs and, in an interview in *the Lancet*, the lead author expressed concern that “we don’t seem to be able to teach them [women] to do breast self examination better. We’re concerned that women will stop being aware of their breasts, and that’s not the message we want to come out of this.” (Larkin, 2001, p. 2109). Thus she suggests that perhaps technique—not procedure—is partly at fault.

However, the “gold standard” study of over 260,000 women by Thomas et al. (2002) reported that the efficacy of conducting BSE for decreasing breast cancer mortality was unproven. One important caveat, as noted by the authors, was that this study was a trial of the teaching of BSE, not of the practice of BSE:

…it should not be inferred from the results of this study that there would be no reduction in risk of dying from breast cancer if women practiced BSE competently and frequently. It is possible that highly motivated women could be taught to detect cancers that develop between regular screenings, and that the diligent practice of BSE would enhance the benefit of a screening program.….. It is, however, unlikely that the level of BSE activity necessary to effect a change in breast cancer mortality could be achieved in a general population of women. ….Until such a trial is conducted, there is no reason to discourage women who choose to practice BSE from doing so. However, it should be emphasized to such women that they must practice BSE regularly and with a high degree of proficiency

(Thomas et al. 2002, p.1456).

If the process is taught correctly, and women are motivated to practice regularly and effectively, perhaps mortality rates may differ. The problem may lie not in the idea, but in its execution, two very different concepts.

## Teaching the Skill of Conducting BSE

The study by Thomas and colleagues emphasizes the importance of teaching BSE as a skill coupled with sufficient levels of motivation to practice the skill regularly. Skills are taught differently than knowledge. Imparting information—basic knowledge transmission—involves *what* to do, but not *how* to do it. Just as learning to drive a car, dance, or play a sport cannot occur solely through listening to a tape or reading a book, conducting a BSE cannot be taught just by telling someone what to do. A Chinese proverb states: *I listen—and forget. I see—and remember. I do—and understand.* When learning skills, the best pedagogical process involves first naming the skill and describing its use and importance. The skill is then demonstrated so that the individual can observe correct and incorrect use of the skill. The modeling is followed by extensive supervised practice of the skill with continuous feedback. Finally, learning a skill involves more than a onetime event; there must be a commitment to continual practice until it becomes an integral part of one’s behavioral repertoire (Danish and Hale, 1981). In other words, learning skills cannot be “caught; ” they must be taught.

### BSE and the Opportunity to Reduce Health Disparities

Modern medicine is driven by economics; health care providers are constantly pressured to see more patients in less time. Physicians and other health care professionals have little time or incentive to teach, rather than tell, their patients how to conduct BSE. Moreover, in areas where health disparities are the greatest, among the urban or rural poor, many lack health insurance. The incidence rate for breast cancer is higher among Caucasian women than African American women, yet more African American women die from the disease. According to the National Cancer Institute’s Web site (2008), factors such as SES play a prominent part in this disparity but do not tell the whole story. African-American women not only seem to have more aggressive tumors than white women, but they are also more likely to delay seeking treatment, and, when they do, they are less likely to receive the most advanced care.

The importance of culture and perception is underscored by study involving more than 1,300 women in New York who had emigrated from Eastern Europe and several Caribbean nations as well as American-born African-American and White women (Consedine, Magai, and Neugut, 2004). Results from this diverse sample suggested embarrassment prevented women from seeking mammograms and clinical breast exams even when controlling for demographic variables. The study did not address BSE, and one advantage of self exams is that it can be conducted alone, thus sparing those who are embarrassed from a CBE, unless they find a lump.

To address this problem, creative solutions are needed. One wave of studies has focused on involving African-American salon employees in the education and teaching of breast health (Sadler et al. 2007; Wilson et al. 2008). Also, Borrayo (2007) found that Latinas face health care barriers ranging from immigration status to language issues to lack of health insurance, and she urges a partnership approach to reach this group of underserved women. For Chinese women, the difference between awareness and practice is great, and simply being aware of BSE is not the same as utilizing it (Wong-Kim and Wang, 2006). When we consider cancer’s effects by socioeconomic status, the news is even grimmer. Research shows that low SES individuals suffer from chronic diseases at a higher rate than their higher-SES counterparts (Kennedy, Paeratakul, Ryan, and Bray, 2007). Preventive care can make a difference to those who lack access to the latest technology. As Chu, Miller, and Spring-field (2007) note, “we can get reductions in disparities in interventions that involve early detection and cancer treatment with all the associated SES issues” (p. 1104).

For the younger generation, especially in underserved communities, schools (nurses and health teachers) may become the default health care system unless a student is very sick or becomes pregnant.

### BRIDGE: Bridging the Gap to Better Health

With the recognition of the increasing role that schools play in health care education, the Life Skills Center at Virginia Commonwealth University developed a health intervention, BRIDGE, for 9th grade boys and girls to teach them how to become their own health historians by having them learn genealogy. Additionally, they were taught other health-related life skills including how to do breast and testicular self-examinations. The program was first pilot-tested in a small rural school. Students were tested prior to the intervention, following the completion of the intervention and three months later (Harmon et al. 2005). Following the pilot-test, an NCI grant funded a larger study.

Training in BSE and testicular self-exams (TSE) was conducted by Hadassah’s “Check it Out!” program. Hadassah is an international Jewish women’s organization that has as one of its missions to teach youth self-examination skills to minimize the effects of cancer. A Hadassah nurse was used to convey skills and information, an American Cancer Society video was used to supplement program material, and a cancer survivor (either breast or testicular as appropriate) spoke about his or her personal story with cancer detection and treatment. Breast and testicular cancer self-examinations were demonstrated on synthetic models and presented as an example of an important component of all cancer prevention. This workshop also discussed the importance of clinical screenings for cancer such as clinical breast examinations, clinical testicular examinations, and mammography. During this process, students were divided by gender.

SAS PROC MIXED was used to analyze the data from the BRIDGE study with schools nested within the treatment condition as a random effect. Unadjusted and adjusted analyses were conducted. Data were analyzed from baseline, immediate post-test and 3-month follow-up for those females who most likely completed all three questionnaires given our linking algorithm. Of the 611 female adolescents who were in 9th grade, 71.4% were white. At baseline, no statistically significant differences in ever performing breast self-exam existed between treatment conditions. After adjusting for age and the baseline measure, at 3-month follow-up, white adolescents in the intervention condition were more likely to report ever performing breast self-exam compared to those in the control condition (62.3% vs. 24.0%, p-value = 0.0009). Similarly, African American adolescents and those of other races in the intervention group were more likely to report ever performing breast self-exam (68.5% vs. 36.1%, p-value = 0.0029). At baseline, adolescents in the intervention condition were more likely to report performing breast self-exam once a month (0.1% vs. 0.0, p-value = 0.0064). After adjusting for age, race, and baseline measures, there were no statistically significant. However, the intervention group was more likely to perform breast self-exam once a month compared to the control group (39.1% vs. 25.6%, p-value = 0.0013) (R.M. Jones, personal communication, May 30, 2008). A skills observation technique was also developed to assess whether the skills of BSE and TSE were learned. The skills observation technique was piloted approximately one year after the pilot-test project was completed with the same sample (Harmon et al. 2005). With regard to the BSE results, 100% of girls in both the control and intervention groups had heard of a self-breast examination and knew what they are used for. In terms of knowledge for the breast self-exam, the average knowledge score for the five girls in the intervention group was 4 out of 6 components correct whereas for the five girls in the control group the average score answered correctly was 1 out of 6. The intervention groups were also better at detecting lumps in the synthetic models, with 100% of the girls able to detect at least one lump. In the control groups, only 20% of the girls were able to detect any lumps. Similar results were found for boys with regard to TSE. (Danish, 2008).

Thus, we see how students can learn these health-related skills. Awareness campaigns are not enough. People need to know not only what to do, but exactly how to do it. Teaching self-directed health care skills is easy, inexpensive, and has no side effects. Claims about straining an already-over-taxed system or the fear of false positives causing excessive worry are legitimate—but if you or a family member is the one case for whom it makes a difference, the risk certainly seems worth the cost. Another indirect benefit is that self-exams provide us with an avenue to really connect with our bodies and provide a source of efficacy in controlling our health destinies.

### (Wo)Man vs. Machine: Self-Exams as the First Step in a Paradigm Shift

To this point, we have been making the case for conducting BSE. It is not altogether clear from the literature whether or how conducting BSE affects mortality rates. Moreover, we can demonstrate with little effort that we can teach high school girls how to conduct a BSE. However, reducing the mortality rate from breast cancer or even reducing the health disparities may be but one benefit of learning how to conduct BSE. While the true costs and benefits of BSE are yet to be determined, the question itself is emblematic of a larger public health concern—that is, in this day and age of mammograms, X-rays, and CAT scans, do women even feel connected enough to their own bodies to believe that they can be trusted to examine them?

In 1970, the Boston Women’s Health Collective published the first edition of *Our Bodies, Ourselves*. The 70s and 80s were a time when the importance of taking care of our own health was stressed. It is an interesting paradox that the more we know about the mechanisms of disease and how to stay healthy, the less we understand what our own bodies are saying to us. For example, walk into any gym and notice the number of treadmill users strapped to heart rate monitors; we know we are supposed to exercise, but we don’t even trust ourselves to know when we are working hard enough. Think back to your last doctor’s visit: when was the last time you had a thorough physical in which the physician felt and listened to your body without the use of sophisticated technology or read-outs from a machine?

None of this, of course, is meant to discount the incredible technological advances we have seen in recent years. Countless lives have been saved and improved because of more sophisticated diagnostic measures. However, as medicine has become more complicated, we have become, to a large extent, controlled by technology and pharmacology. We are exhorted through advertising to take care of ourselves—eat more fruits and vegetables, reduce fats in our diets, don’t smoke, exercise, get exams, use sunscreen—or else, we will die young. The goal is not to take control of our lives; instead, it is to avoid dying prematurely. Being healthy has become synonymous with preventing disease. Moreover, these exhortations are little more than being told what to do, not how to do it. In an analysis of differing recommendations regarding BSE to French and American women, Eisinger et al. (1999) hypothesize that American culture has promoted higher levels of comfort with self-health care—that literally, Americans are more hands-on and more likely to accept BSEs. French culture, they suggest, is more traditional, even paternalistic when it comes to encouraging people to take responsibility for their health. Taking control, however, is exactly what we advocate when we encourage the continued use of BSEs.

In summary, research on the efficacy of BSE in early breast cancer detection remains equivocal. While negative aspects of BSE, such as increased detection of false positives and associated costs to the health care system exist, the potential benefits of BSE should not be overlooked, particularly when one considers significant health disparities in access to health insurance and care. For those individuals who engage in early detection practices, such as BSE, and successfully detect cancer in its early stages, the potential cost is well worth the price.

Findings from the BRIDGE program indicate that skills for proper BSE can be taught with success. Further, research suggests that BSE can be beneficial when exams are conducted competently and frequently. Our attention should focus on improving BSE instruction so that we can teach women *how* to effectively screen for early stages of breast cancer. Such instruction would describe the use and importance of BSE, provide women with a model of correct and incorrect use of the skill, and follow up with supervised practice of the skill and feedback. Educators should highlight the appropriate time of the month to conduct the exam as well as the components of effective BSE. It is also important that we promote regular practice of the skill.

Shame or stigma associated with self-exams is fortunately a thing of the past. We have amazing technology to aid us in understanding that most complex of machines, the human body. Unfortunately, such technologies are not available to everyone, nor are they foolproof. Further, over-reliance on a machine to tell a woman what she may be able to feel with her own hands is yet another way of reducing self-efficacy for control over health outcomes. It is important that we not bypass our own internal mechanisms for understanding when our body is—and is not—functioning properly. Learning how to do a BSE may be a first step in regaining some control over our lives. It is a health-related life skill. It is not enough to be told to take care of ourselves; it is time to learn how to take care of ourselves.
